# Multilayered Composites with Carbon Nanotubes for Electromagnetic Shielding Application

**DOI:** 10.3390/polym15041053

**Published:** 2023-02-20

**Authors:** Povilas Bertašius, Artyom Plyushch, Jan Macutkevič, Jūras Banys, Algirdas Selskis, Oskars Platnieks, Sergejs Gaidukovs

**Affiliations:** 1 Faculty of Physics, Vilnius University, Sauletekio Av. 3, LT-10257 Vilnius, Lithuania; 2 Department of Structural Analysis of Materials, Center for Physical Science and Technology, Sauletekio Av. 3, LT-10257 Vilnius, Lithuania; 3 Institute of Polymer Materials, Faculty of Materials Science and Applied Chemistry, Riga Technical University, P. Valdena 3/7, LV-1048 Riga, Latvia

**Keywords:** electromagnetic shielding, carbon nanotubes, layered structures, laminate, polylactic acid

## Abstract

Bulk polylactic acid (PLA)/multiwall carbon nanotube (MWCNT) composites were prepared and investigated in wide frequency ranges (20 Hz–1 MHz and 24–40 GHz). It was determined that the percolation threshold in bulk PLA/MWCNT composites is close to 0.2 vol.% MWCNT. However, the best microwave dielectric properties and absorption were observed in composites with 3.0–5.0 vol.% MWCNT. Therefore, for future investigations, we selected layered (laminate) polymeric structures with gradual changes in MWCNT concentration from 0.2 to 8.0 vol.% MWCNT. Two approaches to laminate structure designs were examined and compared: a five-layer composite and a nine-layer composite that included four pure PLA middle layers. The addition of MWCNT enhanced the elastic modulus by up to 1.4-fold and tensile strength by up to 1.2-fold, with the best performance achieved at 5.0 vol.% loading. High microwave shielding was observed for these layered PLA/MWCNT structures with a gradient change in MWCNT concentration (up to 26 dB in both transmission and absorption coefficients) in the broad frequency range (from 24 to 40 GHz). Obtained structures are highly anisotropic, and the absorption coefficient is 2–5 dB higher in the direction of MWCNT concentration increase; however, the transmission coefficient is the same in both directions. The properties of microwave absorption are mainly unaffected by the additional polymeric layers. The absorption of the layered structure is greater than the absorption of single-layer composites with an optimal MWCNT concentration of the same thickness. The proposed laminate structure design is promising in the field of efficient electromagnetic shielding.

## 1. Introduction

The rapid growth and diversification of electronic devices and telecommunications are strongly related to a vital pollution problem due to the interference of electromagnetic waves from different sources [1]. This is because different devices are working in the same frequency range. Electromagnetic pollution can destroy electronic devices and harm human health [2]. The pollution problem has become more crucial because, nowadays, a more extensive amount of information is translated via telecommunications channels and, hence, more immense power and higher frequencies of electromagnetic waves are used [3,4]. Electrically conductive metals such as Cu and Al have been traditionally used for electromagnetic shielding applications [5]. The critical drawbacks of metal-based coatings for such applications are the high density, easy corrosion, insufficient flexibility, and large reflected part of electromagnetic radiation. In contrast, coatings based on polymer composites filled with nanocarbon inclusions are lightweight, easily processable, and chemically stable. Therefore, polymeric composites with various nanoinclusions have been suggested for electromagnetic shielding applications [6,7].

Composites with single-wall and multiwall carbon nanotubes (SWCNTs and MWCNTs) have been widely investigated for electromagnetic shielding applications mainly because a very low percolation threshold can be achieved in such systems (less than 0.01 wt.%) [8,9]. Therefore, the required electrical conductivity of composites for various application values can be obtained with a very small amount of these nanofillers. Theoretically, the percolation threshold should be inversely proportional to the radius/length aspect ratio; however, experimentally, the percolation threshold values can be very different for the same type of SWCNT and MWCNT depending on the composites’ preparation technology and the nanotubes’ distribution in the polymer matrix [8,10,11]. For composites with SWCNTs, the microwave transmission strongly decreases with filler concentration, and reflection and transmission coefficients increase and reach very high values (30 dB at 12.4 GHz and thickness 2 mm), mainly due to an increase in conductivity [12,13]. At the same time, the absorption coefficient has a maximum close to some optimal concentration [14]. It was demonstrated that the aspect ratio of carbon nanotubes has an enormous impact on electromagnetic interference (EMI) composites with these inclusions [15,16]. On the other hand, it was demonstrated that the morphological control of the carbon network structure plays a crucial role in their electromagnetic properties [11,17]. The percolation threshold in polymeric composites with other carbon nanoinclusions can also be very low. For example, the electrical percolation threshold in composites with onion-like carbon (OLC) can be as low as 1 vol.%, and, in carbon black composites, it can be less than 0.1% [18,19]. Therefore, it was suggested that polymeric composites with graphene, OLC, exfoliated graphite, and high surface area carbon black could be used for electromagnetic shielding applications [20,21]. Moreover, various hybrid polymeric systems with carbon and magnetic nanoinclusions have also been suggested for electromagnetic shielding applications [22,23].

Polylactic acid (PLA) is a biodegradable and bio-based polymer used as an alternative polymer resin for composite materials with numerous benefits over fossil-based commodity plastics [24]. These advantages include sustainability, comparable mechanical properties, cost-effectiveness, a relatively low melting temperature, compatibility with reinforcement materials, and versatility for use in various industries. The application of PLA in composite processing is an attractive alternative to reduce the dependence on fossil resources and environmental pollution in the production of plastic-based materials. PLA/carbon nanotube (CNT) composites are attracting significant interest as high-performance, multi-functional materials for various industrial applications. Kaseem et al. reviewed potential applications for PLA/CNT composites, which include drug delivery systems, soft and hard tissue engineering, separation membranes, and sensors for liquid sensing and vapor detection [25]. Wang et al. prepared PLA/CNT composites with up to 6.0 wt.% CNT loading and compared them with compositions that used poly(ethylene oxide)-assisted segregated structure [26]. The authors reported that a segregated structure is highly beneficial for higher electrical conductivity; however, the mechanism of EMI shielding is different from that of electrical conductivity. Thus, with higher CNT loading, higher EMI shielding was achieved with just the PLA/CNT combination. Several different approaches for EMI shielding using a PLA matrix have been explored, such as graphene/CNT hybrid nanoparticles [27], supercritical CO_2_ foaming of composites [28], and conductive polymer blends [29]. Nevertheless, to the best of the authors’ knowledge, a laminate design incorporating a multilayer structure approach that involves increasing the CNT gradient has not yet been explored for PLA composites.

It is well known that electromagnetic radiation absorption occurs in a thin layer called the skin depth. Therefore, it is reasonable to use not bulk materials but thin films or porous structures for electromagnetic shielding applications [20]. The best performance of thin films for electromagnetic shielding applications can be achieved when no single thin films are used; however, the multilayer systems of different electrical conductivity layers are employed for electromagnetic shielding applications [30,31,32]. Indeed, in this case, the best compatibility of electromagnetic impedance can be achieved, and the electromagnetic radiation reflected from highly conductive layers can be absorbed in less conductive layers [31,32,33].

This paper aims to prepare a multilayer polymeric structure with an MWCNT gradient structure and explore its electromagnetic compatibility. The proposed concept includes a five-layer system that consists of 0.2, 1, 3, 5, and 8 vol.% of MWCNT-loaded layers. A nine-layer composite incorporating four additional pure PLA middle layers was used for comparison. The selected bio-based and biodegradable PLA matrix incorporates the modern aspects of a sustainable and green eco-design. The results presented with the multilayer gradient structure should be applicable to a wide range of polymeric matrices. 

## 2. Materials and Methods

PLA Ingeo 6201D Natureworks LLC of 1.25 g/cm^3^, with a melt flow index (MFI, at 210 °C) of 15–30 g/10 min, was used. In addition, MWCNT, NC7000 NANOCYL, 9.5 nm, average length 1.5 μm, purity 90%, specific surface 250–300 m^2^/g, volume resistivity 10^−4^ Ωcm, density 2.1 g/cm^3^ was used. Chloroform was provided by Aldrich.

Polymer composites with 0.2–8.0 vol.% of MWCNT were prepared using a solution method (further, all concentrations are volume concentrations). First, PLA and MWCNT were blended in a hot chloroform solution using a 10-min ultrasonic treatment. Then, the obtained blends were dried under the hood at room temperature for 24 h under a 0.05 bar vacuum. 

**Figure 1:**
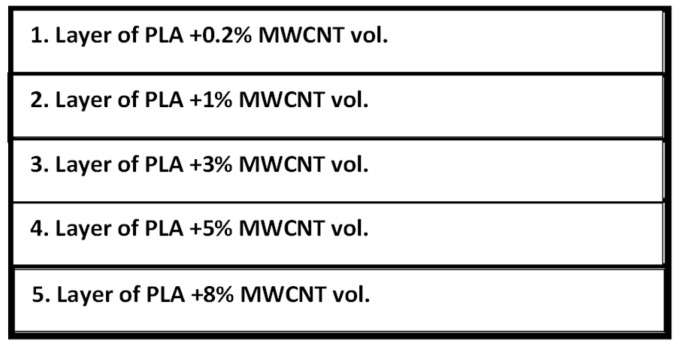

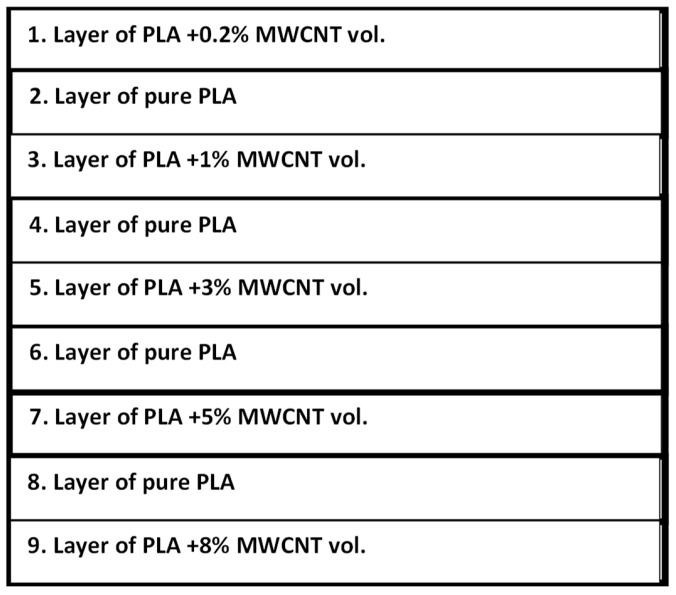
Scheme of investigated layered structures.

The single filler compositions and selected layered structures were obtained through melt molding using Carver CH 4386. The composite materials were molded using compression molding in steel molds. The procedure consists of 2-min preheating, compression for 1 min with 4.5 metric tons pressure at 190 °C, and rapid cooling to 25 °C. The multilayer composites were prepared using the same approach but with molds that have target thicknesses. The obtained layered structures of 5 layers and 9 layers are presented in Figure 1. The thickness of a single layer is about 250 μm.

The tensile properties of bulk composites were investigated using Zwick BDO-FB-020TN equipment according to ISO 527. A load cell of 5 kN with a testing crosshead speed of 2 mm/min was used. The Young’s modulus (E) was calculated from the tangent of the stress–strain curve at the beginning of the coordinate axis. At least five parallel measurements were used for each bulk composite. 

The calorimetric tests were carried out on a Mettler differential scanning calorimetry DSC-1 instrument according to ISO 11357-1. The sample heating rate was set to 10 °C/min, and each sample was heated to 200 °C under nitrogen purge. A sample with a mass of around 10 mg was used. For crystallinity calculations, the melting peak values were used in accordance with the equation:

(1) $$\chi_{c}=\frac{∆H_{m}}{H_{m}^{o}\left(1-W_{MWCNT}\right)}\times 100\%,\;$$

where ∆*H_m_* is the enthalpy of the specimen, ∆$H_{m}^{o}$ is the theoretical melting enthalpy of 100% crystalline polymer (93.7 J/g for PLA), and *W_MWCNT_* is the weight content of MWCNT.

Sartorius KB BA 100 electronic scales equipped with a Sartorius YDK 01 hydrostatic density measurement kit were set up to measure the density (*ρ*) in air and ethanol. The density of the phosphate-buffered saline (PBS) and the composites was calculated using the following equation:

(2) $$\rho =\frac{m_{a}\left(d_{EtOH}-0.00120\right)}{0.99983\left(m_{a}-m_{s}\right)}+0.00120,\;$$

where *m_a_* is the sample’s measured mass in the air; *m_s_* is the sample’s measured mass when the sample is submerged in ethanol; *d_EtOH_* is the density of ethanol, which was measured with the aerometer.

The Mettler Toledo DMA/SDTA861e device was used to measure the thermomechanical properties of the selected samples. Tests were carried out in a dual cantilever measuring system from −50 °C to 100 °C at a heating rate of 3 °C/min in the air with an applied force of 5 N, elongation of 20 μm, and frequency of 1 Hz. The sample dimensions were approximately 80.0 × 10.0 × 1.5 mm.

In the frequency range from 20 Hz to 1 MHz, an LCR meter was used to measure the capacitance and the loss tangent. The transmittance and reflectance in the 24–40 GHz range were measured with a waveguide spectrometer, which includes the generator P2-65 and the scalar network analyzer R2400. For bulk composites, dielectric microwave properties were measured using the thin dielectric road method [34]. For layered structures, plate-like samples were measured.

## 3. Results

### 3.1. Bulk Materials

The tensile and thermal properties and the density of the bulk composite materials are presented in Table 1. An increase in MWCNT concentration in the PLA shows improvements in mechanical properties of up to 5 vol.% of MWCNT. As a result, their ultimate tensile stress (σ) value increased from 44.1 to 52.7 MPa. Accordingly, at the expense of this increase in strength, the values of their modulus of elasticity (E) increased from 819.6 to 1182.9 MPa, and the deformation (ε) at which the ultimate tensile stress is observed (from 7.6 to 5.1%) decreased. Although the tensile strength slightly increased for composites with 3 and 5 vol.% loadings, it is relatively small (0.4 MPa), while the elongation decrease is 0.8%. This indicates that the optimal concentration of MWCNT loading for the highest mechanical properties is between 3 and 5 vol.%. 

Above 5 vol.% filler concentration, the mechanical properties of the composites begin to deteriorate due to the agglomeration of MWCNT, as well as various defects and voids [35]. Therefore, above 5% MWCNT, the composite material shows worse mechanical properties than the pure PLA matrix. The density data shows a gradual increase with MWCNT loading, which coincides with the higher filler density (2.1 g/cm^3^) compared to PLA density (1.25 g/cm^3^). 

From the DSC data, we can observe that the sample’s glass transition temperature (T_g_) shows slight changes in all compositions within the range of 50–54 °C (Table 1). This increase in T_g_ temperature can be related to terminated molecular mobility and structural changes (more compact chain structure and lower free volume) in the amorphous phase of the polymer [36]. 

According to the DSC, the degree of crystallinity (X_c_), which corresponds to the amounts of the crystalline phase of the polymer matrix, is affected by the MWCNT concentration. The MWCNT hinders the macromolecular chain mobility and acts as a nucleating agent for growing the polymer crystals [35]. In addition, even a small change in nanoparticle loading can yield significant structural changes in the polymer’s crystalline structure. While this is not the focus of this research, it could contribute to the scattering of crystallinity values. The variations in the nanostructure of composites could produce synergetic or complementary relationships between layers, thus enhancing EMI shielding compared to fixed-concentration bulk samples. The increase in MWCNTs in the polymer matrix does not significantly change the material’s melting point (T_m_; T_m_ was in the range from 164 to 166 °C).

**Figure 2:**
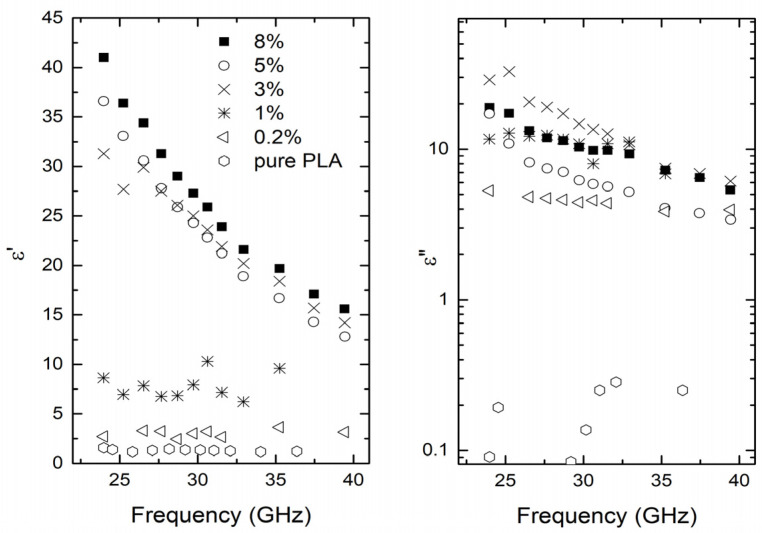
The measured real (**left**) and imaginary (**right**) part of the complex dielectric permittivity in a 24–40 GHz frequency range of CNT/PLA composites.

The complex dielectric permittivity of samples with different concentrations of MWCNT inside a PLA matrix in a 24–40 GHz frequency range is presented in Figure 2. The MWCNT electrical percolation effect was observed with a threshold value close to 0.2 vol.%, resulting from the sharp increase of both real and imaginary parts of dielectric permittivity values and frequency independent conductivity values for MWCNT concentrations not less than 0.2 vol.% in the frequency range 20 Hz–1 MHz (Figure 2 and Figure S1). The complex dielectric permittivity decreases with frequency in good agreement with Jonsher universal law [13,37]. At higher MWCNT concentrations (not less than 3 vol.% MWCNT), the dielectric permittivity and dielectric losses are almost concentration-independent, which is typical for composites above the percolation threshold [38].

However, the best microwave properties are observed for composites with 3–5 vol.% MWCNT. For example, for composites with 8 vol.% MWCNT, the dielectric permittivity is 28, while dielectric losses are 10 at 30 GHz. According to calculations performed in [39,40], such dielectric properties correspond to 50% absorption of a 1-mm plate sample.

### 3.2. Layered Structures

**Figure 3:**
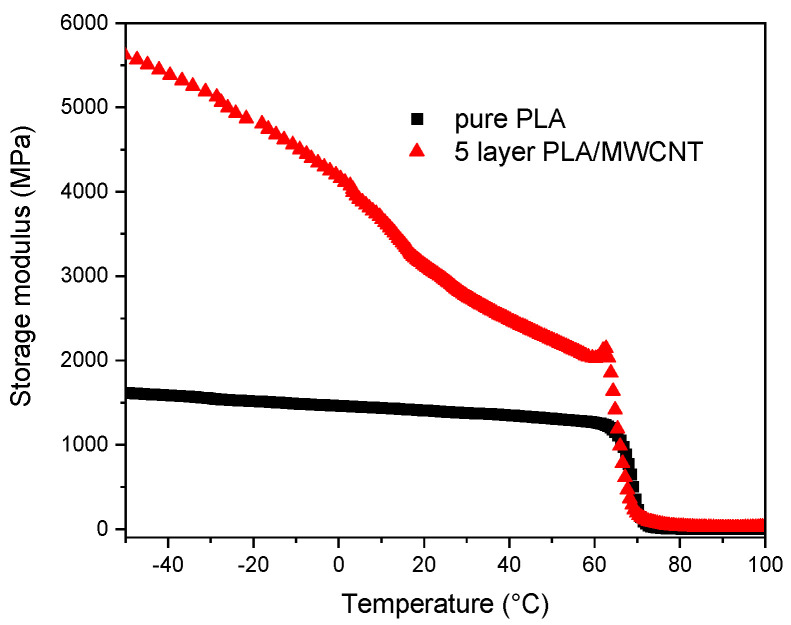
Storage modulus curves of pure PLA and five-layer PLA/MWNCT composite.

A thermomechanical analysis was performed to characterize laminate durability and examine the effective temperature range for the application. Figure 3 shows the storage modulus values for pure PLA and a five-layer PLA/MWCNT composite in the range from −50 to 100 °C. The sharp drop in storage modulus values represents the glass transition region, which is about 10 °C higher than the values obtained using DSC. This can be explained by the difference in measured sample mass, heating rate, and measurement method. After the glass transition, PLA becomes a relatively soft material, losing most of its stiffness. Thus, composites retain their dimensional stability up to around 60 °C. However, there is a large gap between the composite and PLA performances in the glassy state. There is also a much more pronounced drop in the storage modulus values in the glassy state for composites that experience an increase in temperature compared to PLA. This could be attributed to decreased intermolecular bonding with an increase in temperature, which could affect the load distribution between the layers. Overall, the five-layer composite showed a significant increase in storage modulus compared to PLA, indicating the formation of a uniform composite with good load distribution and energy transfer capabilities. 

**Figure 4:**
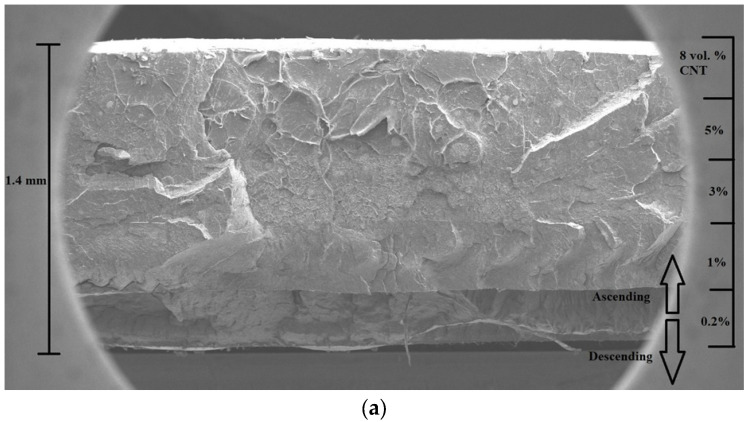

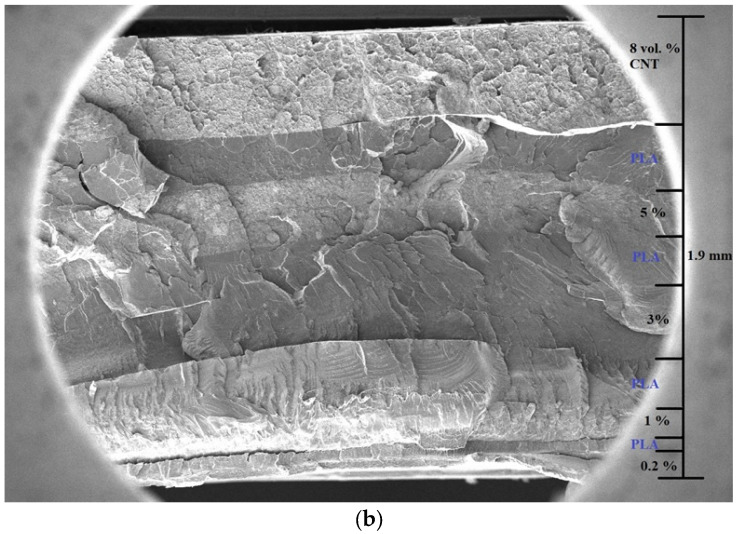
SEM pictures of layered structures (**a**) without an additional polymer layer, (**b**) with an additional polymer layer.

Scanning electron microscope (SEM) images of layered structures are presented in Figure 4. It shows the layered structure of samples, while the distribution of the MWCNT in layers is relatively homogenous. The five composite layers fused during the thermal molding process developed a homogenous structure. At the same time, the nine-layered composite developed strong heterogeneous structures with a visible interface between the individual layers of the laminate composites. In addition, it can be observed that higher MWCNT loading in the composites contributed to a more complex (rougher) fracture surface. 

For the nonhomogenous samples, as in the case of the layered structures of various MWCNT concentrations, the dielectric permittivity measurements using the rod in a waveguide method are impossible. Consequently, only the electromagnetic compatibility measurements of the samples were performed. The incident and transmitted electromagnetic waves in a vector network analyzer are represented by *S*-parameters *S*_11_ (the input port voltage reflection coefficient) and *S*_12_ (the reverse voltage gain), respectfully, with the transmission (T) coefficient being equal to the reflection ^®^ coefficient, expressed as follows (more information about *S* parameters is provided in [41]):

(3) $$R={\left|S_{11}\right|}^{2},$$

(4) $$T={\left|S_{12}\right|}^{2}.\;$$

The transmission coefficient is the ratio between incoming ($P_{i}$) and outgoing power ($P_{t}$) and, consequently, the total EMI SE is obtained using:

(5) $$SE_{T}\;\left(dB\right)=\;-10\;log\left(T\right)=SE_{A}+SE_{R}+SE_{M},$$

(6) $$T=P_{i}/P_{t}.\;$$

Here, the $SE_{A}$ is the absorbed power inside the material, $SE_{R}$ is the power lost due to reflection, and $SE_{M}$ are multiple reflections inside the material, which can be neglected since it is close to 0 when $SE_{A}$ > 10 dB. The absorption coefficient (*A*) is expressed as A=1−T−R. To calculate the effective absorbance, $(A_{eff}$) needs to be calculated concerning the incident power on the sample:

(7) $$A_{eff=}\left(1-T-R\right)/\left(1-R\right).$$

Consequently, $SE_{A}$ and $SE_{R}$ are expressed as:

(8) $$SE_{R}=-10\;log\left(1-R\right),\;$$

(9) $$SE_{A}=-10\;log\left(1-A_{eff}\right).\;$$

The reflection loss *SE_R_* for multilayered structure can be expressed as [13]

(10) $$SE_{R}=20\;log(\frac{1}{2}\left|1+\frac{Z_{1}}{Z_{0}}\right|)+20\;log(\frac{1}{2}\left|1+\frac{Z_{2}}{Z_{1}}\right|)+\ldots +20\;log(\frac{1}{2}\left|1+\frac{Z_{n+1}}{Z_{n}}\right|)$$

where *Z_n_* is the impedance of materials. Thus, the reflection losses are lower when the ratio *Z_n_*_+1_/*Z_n_* is minimal. On the other hand, the *SE_A_* of the multilayer composites can be approximated as the sum of the absorption in each layer. For example [42]:

(11) $$SE_{A}=8.686(\frac{d_{1}}{\delta_{1}}+\frac{d_{2}}{\delta_{2}}\;+...+\frac{d_{n}}{\delta_{n}})$$

where *d_n_* is the shielding thickness and *δ_n_* is the skin depth of materials.

**Figure 5:**
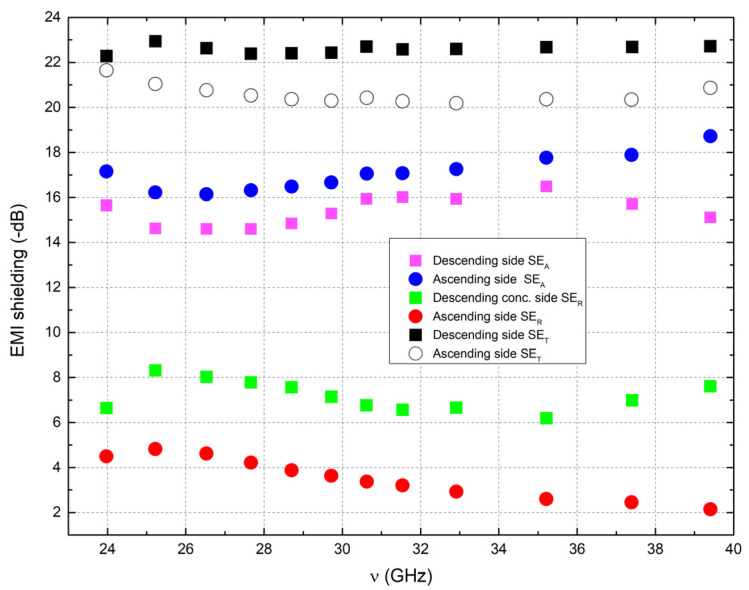

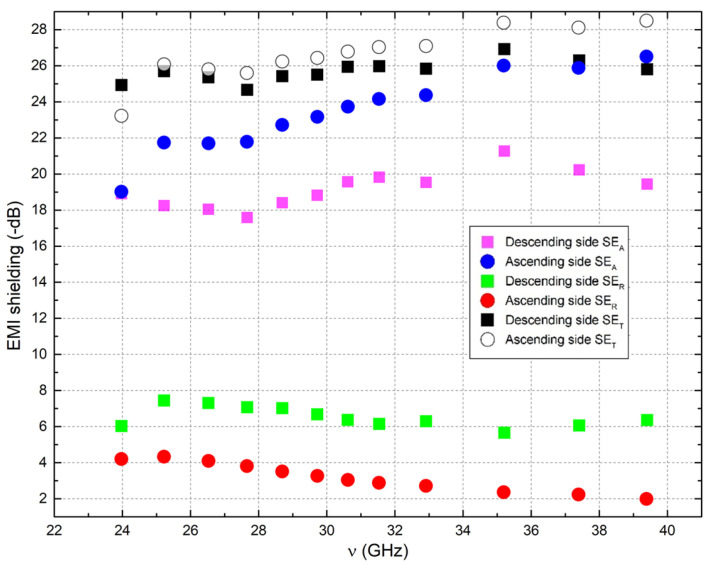
The EMI SE results for the MWCNT/PLA-layered composites (top without additional polymer layer, bottom with additional polymer layer) in the 24–40 GHz frequency range.

The measured electromagnetic compatibility of the 1.45-mm layered composites in a comprehensive 24–40 GHz frequency range is presented in Figure 5. The sample was measured both from the ascending (0.2 vol.% to 8 vol.%) and the flipped descending side (8 vol.% to 0.2 vol.%). Overall, the sample showed good EMI SE properties, with the $SE_{R}$ falling below the value of 8 dB and $SE_{T}$ being above the value of 18 dB in the whole frequency range. The value of $SE_{A}$ is close to 15 dB in the whole range. When the descending side was in contact with the incident wave first, there was a reduction in $SE_{R}$ by about 2 dB in the whole frequency range.

Consequently, the $SE_{A}$ was increased by about 1–2 dB while the $SE_{T}$ remained the same regardless of which side was used. This effect can be explained by reducing the total impedance (the ratio of transverse components of the electric and magnetic fields’ mismatch of air and layers; the impedance of two media must match to avoid reflections [42]) since the high conductivity material is gradually presented to the electromagnetic wave.

The absorption of the layered structure is more significant than the absorption of single composites with an optimal MWCNT concentration (Figure 2) layer with the same thickness [34,35]. 

## 4. Conclusions

This work explores the concept of multilayer polymeric structures with an MWCNT gradient laminate design. The electromagnetic shielding efficiency of the single layer (bulk) composites are assessed for optimal filler concentration as an alternative to the multilayer laminate approach. In addition, a laminate composite with an incorporation of pure middle layers is explored. The mechanical properties showed the highest elastic modulus and tensile strength for a 5 vol% loaded MWCNT composite, with a 1.4- and a 1.2-fold increase, respectively. Thermomechanical testing reported a five-layer composite storage modulus increase over the pure PLA. In addition, MWCNT contributed to a significant increase in the storage modulus in the measured temperature range (especially in the glassy state). 

The prepared bulk PLA/MWCNT composites were investigated in a wide frequency range. It was determined that the percolation threshold in bulk PLA/MWCNT composites is close to 0.2% MWCNT. However, the best microwave dielectric properties and absorption were observed in composites with 3–5 vol.% MWCNT. Therefore, the investigations focused on layered polymeric structures with gradual changes in MWCNT concentration from 0.2% to 8% MWCNT. High microwave shielding was observed for these layered PLA/MWCNT structures with a gradient change in MWCNT concentration (up to 26 dB in both *SE_T_* and *SE_A_*) in the broad frequency range (from 24 to 40 GHz). Obtained structures are highly anisotropic, and the absorption coefficient is 2–5 dB higher in this direction when MWCNT concentration increases due to the better impedance mismatch. However, the transmission coefficient is the same in both directions. The additional polymeric layer minimally affects microwave absorption properties.

PLA and other bio-based and biodegradable polymeric matrices continue to contribute to a growing number of publications and research interests. Our findings evidence the viability of a transition from fossil-based electromagnetic plastics to more sustainable electromagnetic biobased polymeric materials. The proposed PLA-based EMI materials have very competitive results compared to fossil-based polyolefin solutions. The multilayer gradient structure presented in this work should be applicable to a wide range of polymers; however, further durability research on electromagnetic application validation is necessary.

## Figures and Tables

**Table 1:** Physical properties of the bulk materials.

Sample	(g/cm^3^)	T_g_ (℃)	T_m_ (°C)	X_c_ (%)	E (MPa)	σ (MPa)	ε (%)
0%	1.254	50	165	34	819.6 ± 32.5	44.1 ± 6.9	7.6 ± 0.7
0.2%	1.261	54	165	42	847.4 ± 66.9	48.1 ± 4.1	6.9 ± 1.6
1%	1.266	53	164	38	892.0 ± 14.8	50.9 ± 0.2	6.8 ± 0.1
3%	1.282	53	166	42	1024.9 ± 41.0	52.3 ± 7.3	5.9 ± 0.5
5%	1.292	52	164	40	1182.9 ± 66.2	52.7 ± 2.6	5.1 ± 0.8
8%	1.310	54	165	45	879.3 ± 140.4	39.9 ± 12.1	4.8 ± 0.5

## Data Availability

Not applicable.

## Supplementary Materials

The following supporting information can be downloaded at: https://www.mdpi.com/article/10.3390/polym15041053/s1, Figure S1: The measured dielectric permittivity (left) and electrical conductivity (right) in a 20 Hz–1 MHz frequency range of CNT/PLA composites.
