# Supplementary material for: Multilayered Composites with Carbon Nanotubes for Electromagnetic Shielding Application
Source: Polymers (Basel). 2023 Feb 20;15(4):1053. doi: 10.3390/polym15041053 (PMC9963311; doi:10.3390/polym15041053)
Supplement: Supplementary file 1 [file polymers-15-01053-s001.zip › polymers-2149658-supplementary.pdf]

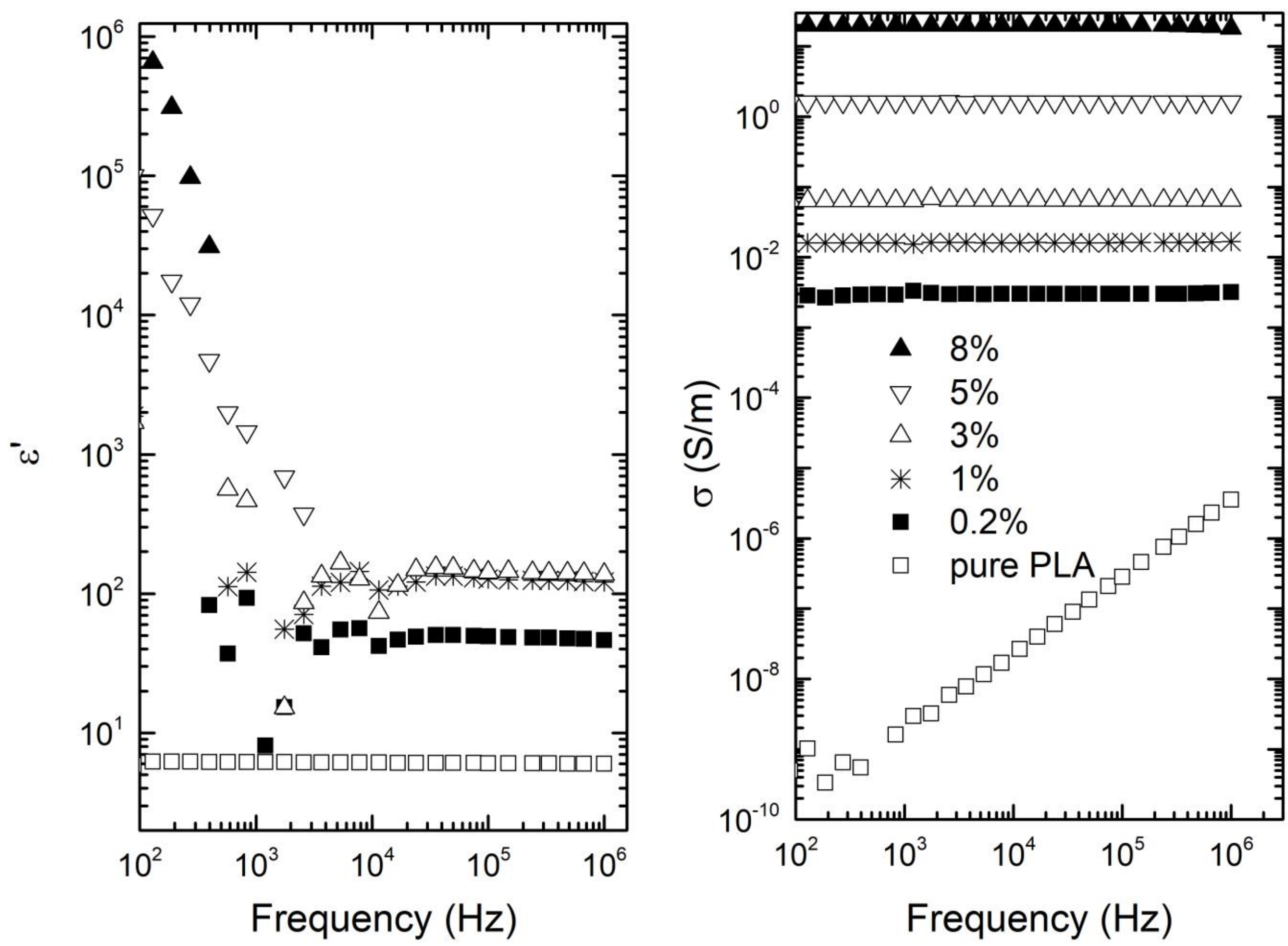

Figure S1. The measured dielectric permittivity (left) and electrical conductivity (right) in a 20 Hz - 1 MHz frequency range of CNT/PLA composites.
